# Commercial farmed swine harbour a variety of pathogenic bacteria and antimicrobial resistance genes

**DOI:** 10.1099/jmm.0.001787

**Published:** 2024-01-17

**Authors:** Thanaporn Eiamsam-ang, Pakpoom Tadee, Songphon Buddhasiri, Phongsakorn Chuammitri, Nattinee Kittiwan, Ben Pascoe, Prapas Patchanee

**Affiliations:** 1 Graduate Program in Veterinary Science, Faculty of Veterinary Medicine, Chiang Mai University, Muang, Chiang Mai, Thailand; 2 Veterinary Academic Office, Faculty of Veterinary Medicine, Chiang Mai University, Muang, Chiang Mai, Thailand; 3 Veterinary Research and Development Center (Upper Northern Region), Hang Chat, Lampang, Thailand; 4 Centre for Genomic Pathogen Surveillance, Pandemic Sciences Institute, University of Oxford, Oxford, UK; 5 Ineos Oxford Istitute for Antimicrobial Research, Department of Biology, University of Oxford, Oxford, UK

**Keywords:** antimicrobial resistance (AMR), caecal microbiome, commercial swine farming, shotgun metagenomics, swine

## Abstract

**Introduction.** The northern region of Thailand serves as a crucial area for swine production, contributing to the Thai community food supply. Previous studies have highlighted the presence of foodborne bacterial pathogens originating from swine farms in this region, posing a threat to both human and animal health.

**Gap statement.** Multiple swine bacterial pathogens have been studied at a species level, but the distribution and co-occurrence of bacterial pathogens in agricultural swine has not been well established.

**Aim.** Our study employed the intestinal scraping technique to directly examine the bacterial micro-organisms interacting with the swine host.

**Methodology.** We used shotgun metagenomic sequencing to analyse the bacterial pathogens inhabiting the caecal microbiome of swine from five commercial farms in northern Thailand.

**Results.** A variety of pathogenic and opportunistic bacteria were identified, including *Escherichia coli*, *Clostridium botulinum*, *Staphylococcus aureus* and the *Corynebacterium* genus. From a One Health perspective, these species are important foodborne and opportunistic pathogens in both humans and agricultural animals, making swine a critical pathogen reservoir that can cause illness in humans, especially farm workers. Additionally, the swine caecal microbiome contains commensal bacteria such as *Bifidobacterium*, *Lactobacillus* and *Faecalibacterium*, which are associated with normal physiology and feed utilization in healthy swine. Antimicrobial resistance genes were also detected in all samples, specifically conferring resistance to tetracycline and aminoglycosides, which have historically been used extensively in swine farming.

**Conclusion.** The findings further support the need for improved sanitation standards in swine farms, and additional monitoring of agricultural animals and farm workers to reduce contamination and improved produce safety for human consumption.

## Introduction

The northern region of Thailand serves as a crucial area for swine production, contributing to the Thai community food supply [1,2]. In 2022, swine production, export and domestic consumption in Thailand were 1.45 million, 5,676, and 1.15 million tons, respectively. The pork industry in Thailand is vital for protein and meat production, with quality-control standards beginning at the farm level [3]. Furthermore, northern swine farms have diverse farming types and farm scales [4]. Previous studies have highlighted the presence of foodborne pathogens originating from swine farms in this region using whole-genome sequencing analysis [5]. Gut microbiomes include the full complement of micro-organisms that live in the digestive tracts of living animals [6] and diversity in the gut microbiome reflects variation in the host species, which can be influenced by farming conditions, environment and antimicrobial usage [7,9].

Culture-independent shotgun metagenome sequencing can yield data on the co-occurrence of bacterial species [9,10]. By comparing information on the microbial diversity of the gut microbiome, antimicrobial resistance genes and genes involved in other biological and metabolic processes, we can better investigate the relationship between microbes and swine [8,11]. Furthermore, this technique can be used to trace pathogen transmission from the swine gastrointestinal tract to humans, encompassing One Health principles [12,14]. Investigations of differences in the microbiomes of livestock animals have included studies on the impact of antimicrobial growth promoters on microbial abundance and metabolic profiles in poultry [15], and altered nutrient utilization and secondary metabolic production in bovines [16].

In swine, shotgun metagenome analyses have also been applied to investigate the antimicrobial and heavy metal resistance genes harboured in fattening swine caecal microbiomes, as well as the effect probiotic supplementation has on piglet health [17,18]. While previous studies have shown that caecal microbiomes are diverse and can be correlated with high feed efficiency and utilization [9,19], few studies have utilized the intestinal scraping technique to study micro-organisms directly interacting with the host, particularly in swine [20,21]. In this study, a shotgun metagenome approach was implemented to thoroughly investigate and characterize the pathogenic and commensal bacteria harboured in the swine gut microbiome. Additionally, the intestinal scraping technique was used to directly examine bacteria that specifically attach and interact with swine caecal epithelial cells from commercial farms in the northern region. This methodology facilitated the acquisition of a comprehensive overview of the swine caecal microbiome emphasizing on the analysis of bacterial diversity and genes associated with metabolism, given their direct impact on the well-being and health of the host. Furthermore, our examination extended to the exploration of antimicrobial resistance genes. Understanding bacterial transmission within agricultural systems and the environment helps inform risk to global public health.

## Methods

### Swine caecal sampling

**Fig. 1.**
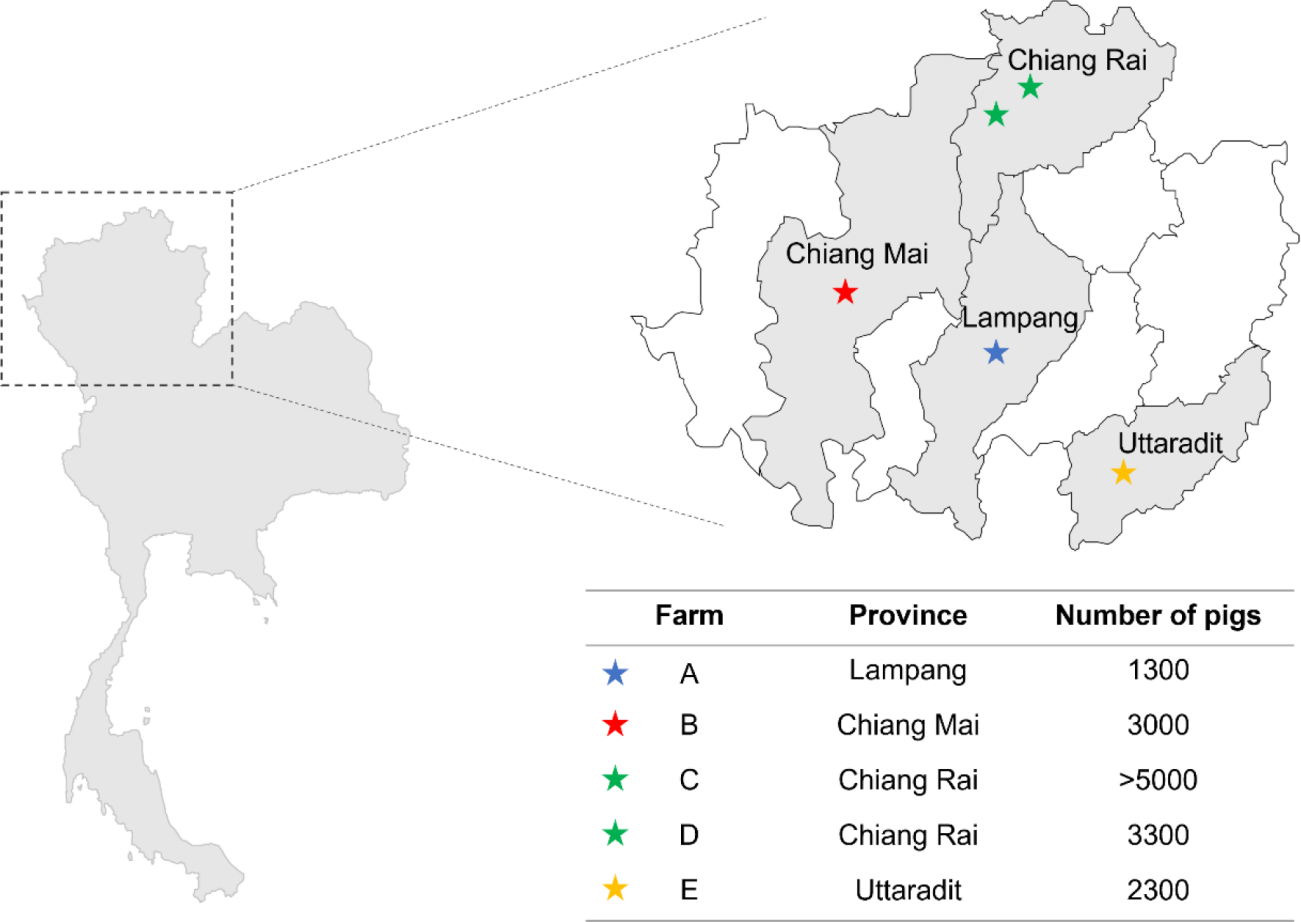
Geographical distribution and the details of five commercial swine farms in this study. All samples were collected from farms located in the upper northern region of Thailand, including Lampang (farm A), Chiang Mai (farm B), Chiang Rai (farms C and D) and Uttaradit (farm E) provinces. All farms represented intensive commercial farms that raised between 1,000 to more than 5,000 pigs per farm.

Five swine caecal samples from five intensive commercial swine farms in northern Thailand were included in this study. All samples were collected from farms located in the upper northern region of Thailand, including Lampang (farm A), Chiang Mai (farm B), Chiang Rai (farms C and D) and Uttaradit (farm E) provinces. All farms represented intensive commercial farms (contains between 1000 to more than 5000 pigs per farm) (Fig. 1). The market-weighted swine from five commercial farms were slaughtered at 120 days of age. The caeca were collected during the period March 2021 through July 2021 at the slaughtering process and stored at −30 °C. Subsequently, the caecal apex was then opened, and a sterilized blade was employed for intestinal scraping technique. The caecal contents were carefully gathered from the mucosal surface with the purpose of studying micro-organisms that directly interact with the host [20,22].

### DNA extraction and shotgun metagenomic sequencing

Genomic DNA was extracted from caecal tissues using a Tissue Genomic DNA Extraction Kit (Qiagen). UV-Vis spectrophotometry and 1 % agarose gel electrophoresis were used to determine DNA concentration and quality. Paired-end library preparation with a read length 150 bp was performed using the TruSeq Nano DNA Kit (Illumina), and libraries were sequenced on the Illumina NovaSeq 6000 platform. The total reads obtained from the metagenomic sequencing was 138,868,453 reads (an average of 27,773,691 reads per sample) (Table S1, available in the online version of this article). Data can be accessed at the National Center for Biotechnology Information with accession number PRJNA937456 (individual accession numbers in Table S2).

### Data quality control and assembly

Sequencing reads were first screened by FastQC (version 0.11.9) for quality assessment [23] and CutAdapt used to trim low-certainty bases and poor-quality reads [24]. Reads were mapped to the swine reference genome (Sscrofa11.1; BioSample ID SAMN02953785) to remove host genome contamination using bowtie2 (v2.3.3) and Samtools (v.1.12) [25,26]. Short reads were assembled *de novo* into contigs with metaSPAdes using *k*-mer sizes of 21, 33, 55 and 77 [27]. Contigs were binned using an expectation-maximization algorithm in MetaBAT2 with a minimum contig length of 1500 [28]. MetaGeneMark was used to predict potential ORFs [29]. Gene sequences with less than 100 bp in length were removed, and remaining sequences were translated into the corresponding amino acid sequences. All predicted genes were clustered, and redundancy was eliminated using CD-HIT (identity > 95 %, coverage > 90 %) [30].

### Taxonomic classification of the swine caecal microbiome

The taxonomic classification of the assembled contigs was performed using Kraken2 against a standard database with the default parameters [31]. The estimate of relative abundance from Kraken2 was determined using Bracken [32]. The taxonomic abundance of bacterial composition in each sample was visualized as the balloon plot and the 100 % stacked bar graph by ggpubr and ggplot2 packages in R [33].

### Functional annotation and profiling of the swine caecal microbiome

For functional annotation of the swine caecal microbiome, putative amino acid sequences of five swine caecal metagenomic contigs acquired from MetaGeneMark were functionally annotated and profiled according to Kyoto Encyclopedia of Genes and Genomes (KEGG) databases using GhostKOALA [34]. Obtained KEGG Orthology were reconstructed to the KEGG pathway and classified into metabolic pathway at 3 levels. The visualization of the KEGG categories and the KEGG pathway in each level were performed using the balloon plot and the 100 % stacked bar graph by ggpubr and ggplot2 packages in R [33].

### Identification of antimicrobial resistance genes

Assembled contigs were used to detect antimicrobial resistance genes harboured by isolates in the swine caecal microbiome using ABRicate against the CARD database (CARD: The Comprehensive Antibiotic Resistance Database) with DNA identity > 85 % and coverage >80 % [35,36]. The detected antimicrobial resistance genes (ARGs) were visualized as a binary heatmap and a Venn diagram, which were constructed using heatmaply and ggplot2 packages in R [33].

## Results

### Bacterial taxonomic diversity in swine caecal microbiomes

Five intensive commercial swine farms were selected from the upper northern region of Thailand to provide an overview of the bacterial micro-organisms present in the digestive tract of swine in the intensive farming system (Fig. 1). Caecal samples from different provinces were extracted and sequenced using a shotgun metagenome approach on an Illumina Novaseq 6000 platform, which generated an average of 27,112, 029 quality filtered reads per sample. After host genomic removal, the remaining reads were an average of 9,221,845 reads per sample. Each individual sample’s sequence reads were assembled *de novo*. The number of contigs ranged from 65, 294 to 229, 191 contigs (Table S1). The assembled contigs were used to classify the bacterial taxonomic composition of each swine caecal sample. In addition to the bacterial composition data in the swine caecal microbiome, taxonomic classification using Kraken2 also revealed the proportions of bacteria, viruses and archaea in each sample (Fig. S1). Comparatively few contigs could be assigned to non-bacterial microbial pathogens on four of the five farms from which we collected samples. While nearly 20 % from farm B could be attributed to viral sources, most of those contigs were characterized as bacteriophage.

**Fig. 2.**
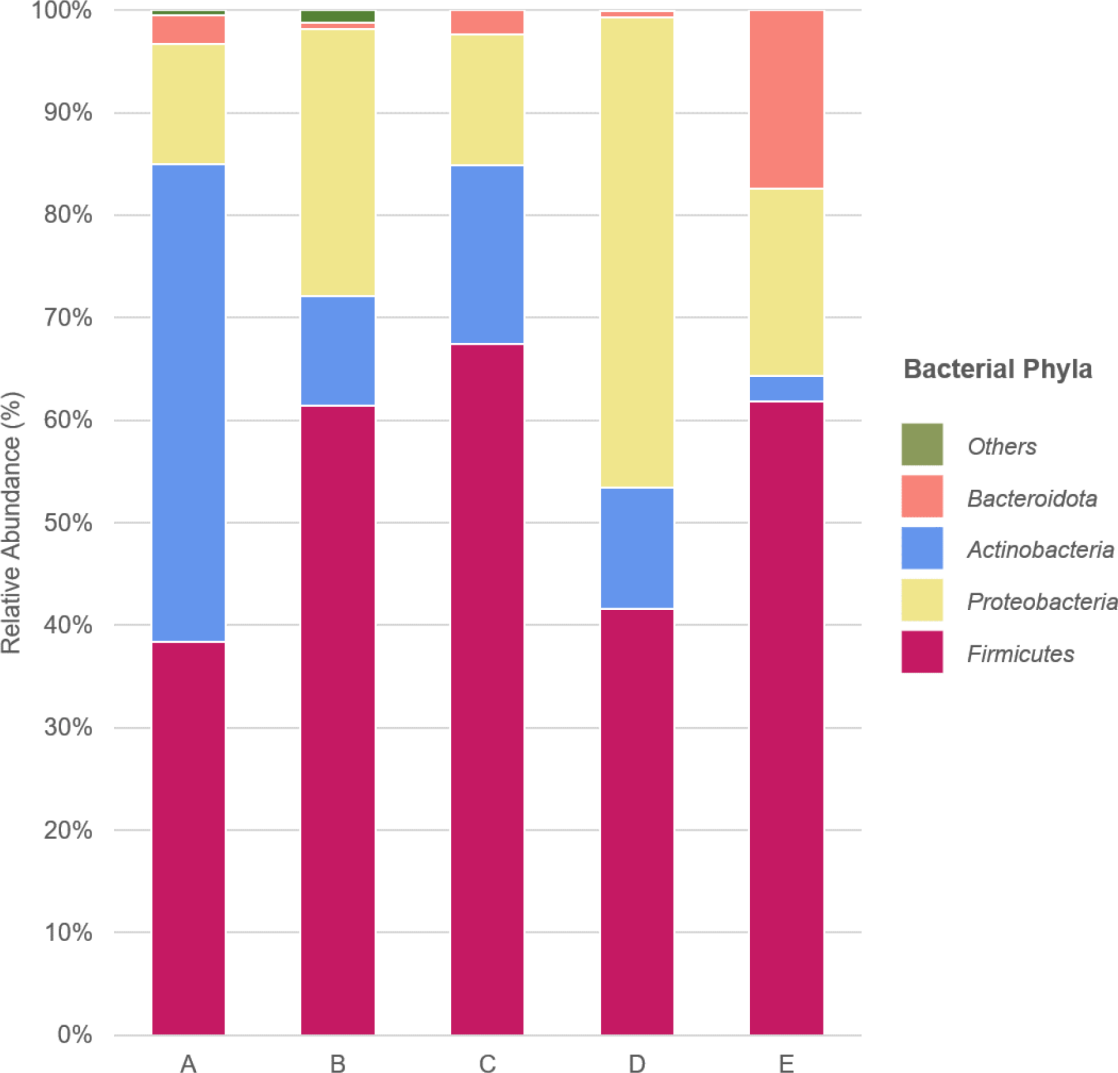
Stacked bar chart of the relative abundance of major bacterial phyla identified on each farm.

Based on the relative abundance, the bacterial phylum-level composition was diverse among the samples. The *Firmicutes* were the most prevalent phyla in swine caeca from farms B, C and E; while *Actinobacteria* constituted the most common phylum in swine caeca from farm A and *Proteobacteria* was the most common phylum in swine caeca from farm D. Farms C and D were located in the same province (Chiang Rai province), but the relative phylum-level composition in swine caeca was different, with farm C containing a higher abundance of *Firmicutes* and *Bacteroidota* phyla than farm D. In contrast, swine caeca from farm D were comprised with a higher proportion of *Proteobacteria* phyla than farm C. Bacterial phyla identified in swine caeca from farm E were the most distinct compared with other farms. Farm E was comprised of a higher proportion of *Bacteroidota* and a lower proportion of *Actinobacteria* than other farms (Fig. 2).

**Fig. 3.**
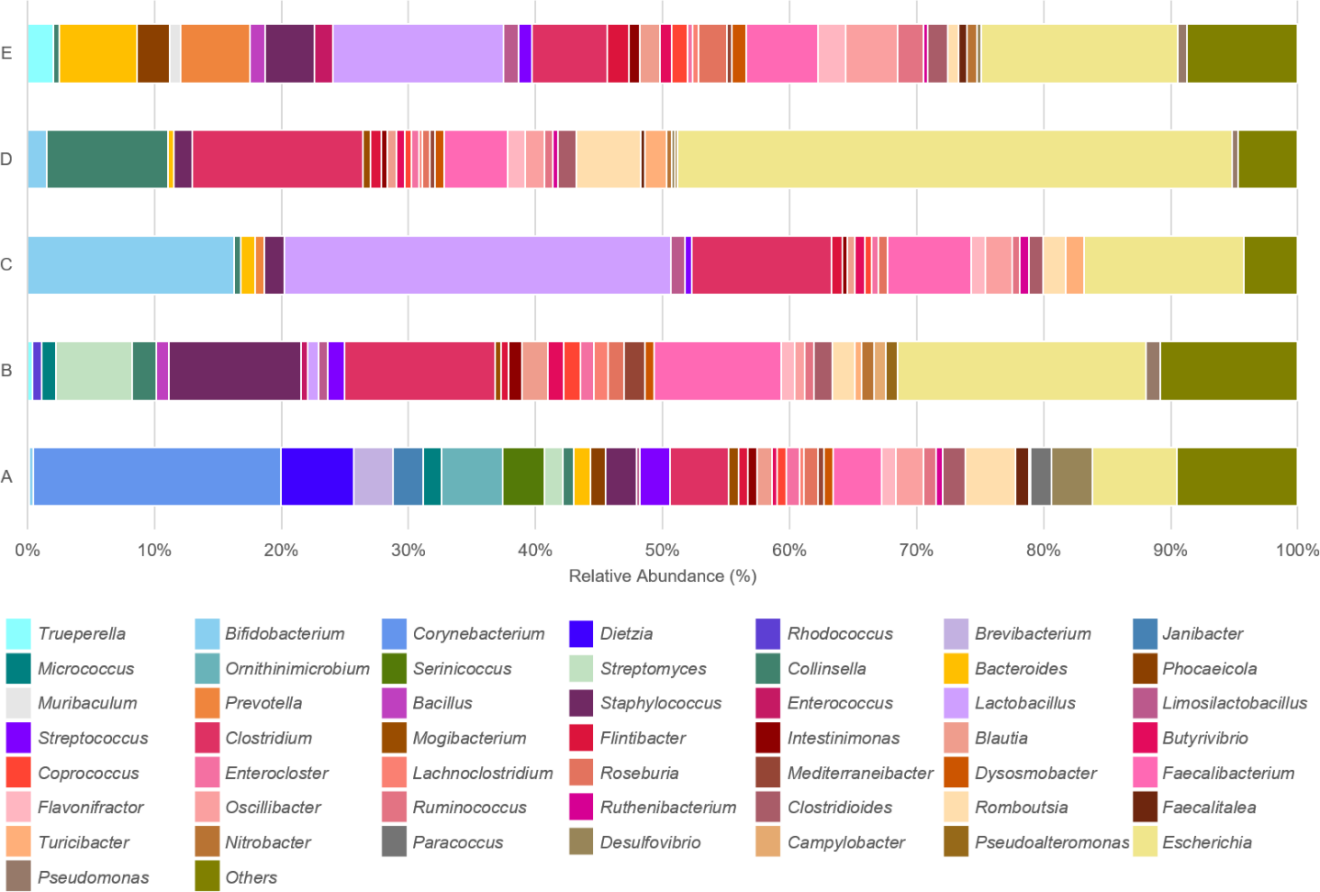
Stacked bar chart of the relative abundance and diversity of major genera identified in swine caecal microbiomes on each farm.

Considerable diversity was also observed in the relative proportion of bacterial genera identified in swine caeca from each farm. *Escherichia*, *Clostridium* and *Faecalibacterium* were the most common genera identified in caeca from all farms. Different bacterial genera were dominant in caeca from different farms; *Corynebacterium* was the dominant genera found in caeca from farm A, followed by *Escherichia. Escherichia* was the most common genus found in caeca from farm B, followed by *Clostridium, Staphylococcus* and *Streptomyces. Lactobacillus* and *Bifidobacterium* were the most common genera in caeca from farm C and *Collinsella* was the predominant genera found in caeca from farm D. Both *Lactobacillus* and *Escherichia* genera were found in the same proportion in caeca from farm E, where the *Bacteroides* and *Prevotella* genera (both *Bacteroidota* phyla) were also common (Figs 3 and S2).

**Fig. 4.**
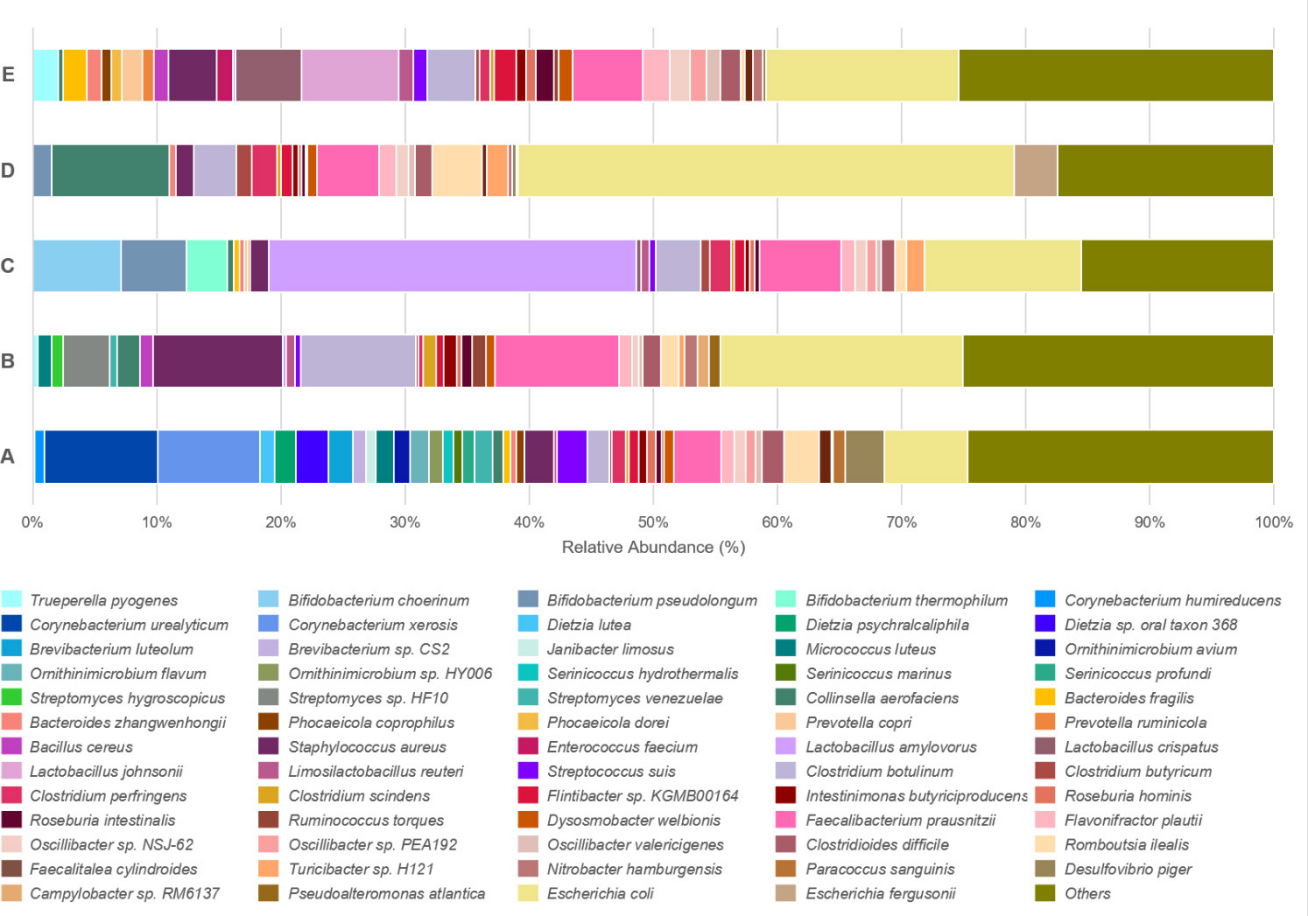
Stacked bar chart of the relative abundance and diversity of bacterial species identified in swine caecal microbiomes on each farm.

At the species level, swine caeca from each farm contained a diverse range of pathogenic, opportunistic and commensal bacteria. *Escherichia coli*, *Clostridium botulinum* and *Faecalibacterium prausnitzii* were the predominant species in all samples. Similar to the genera-level composition, there are some predominant bacterial species found in each individual farms including, *Corynebacterium urealyticum* and *Corynebacterium xerosis* in caeca from farm A while *Streptomyces hygroscopicus* and *Streptomyces* sp*. HF10* were the predominant species and found only in caeca from farm B. Correlated with the genera level, probiotic species *Lactobacillus amylovorus, Bifidobacterium choerinum, Bifidobacterium pseudolongum* and *Bifidobacterium thermophilum* were the predominant species in caeca from farm C. Different species from the genus *Lactobacillus* were the most common in caeca from farm E and C microbiomes; the predominant species in caeca from farm E was *Lactobacillus crispatus* and *Lactobacillus johnsonii*, which differed to the *Lactobacillus* species found in farm C. In farm D, *Collinsella aerofaciens* was the predominant species, followed by *Escherichia coli* (Figs 4 and S3).

### Similarity in functional characterization of swine caecal microbiome genes identified between farms

The predicted genes were functionally annotated using the Kyoto Encyclopaedia of Genes and Genomes (KEGG) databases to assess the potential functional capacity of the swine caecal microbiomes. In total, 3.8–12.3 % of the predicted genes were assigned to the KEGG Orthology and reconstructed to the KEGG pathway (Table S1). All swine caecal microbiome samples contained genes from all four main pathway categories. Genes from the KEGG pathway categories correlated with metabolism were the most commonly identified genes on all farms, followed by genetic information, environmental information and cellular processes categories. There is quite a similar proportion of the relative abundance of the KEGG pathway genes identified between farms (Fig. S4). According to the KEGG pathway at level 2, carbohydrate metabolism was the most prevalent metabolic pathway, followed by amino acid, energy and nucleotide metabolism. Genes identified in microbiome samples from all farms included those with roles in replication, repair and translation associated with the genetic information process. Furthermore, gene-related with environmental information and cellular processes, including membrane transport, signal transduction and cellular community-prokaryotes were also found among all samples (Fig. S4).

**Fig. 5.**
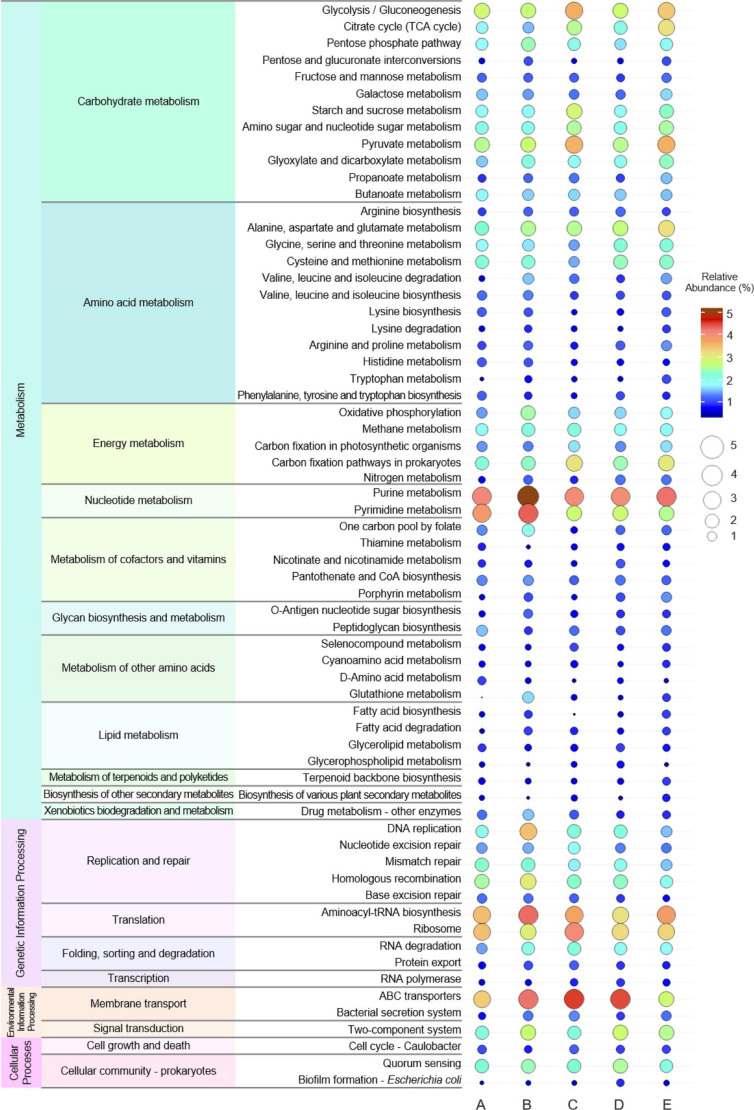
Balloon plot of the functional characterization of the swine caecal microbiome according to the KEGG annotation pathway database at level 3. Circle sizes and colour represented the percentage of the relative abundance in each KEGG metabolic pathway.

Mining these genes at different KEGG Pathways help understand some of the specific metabolic processes that are important in the swine microbiome. Detailed descriptions of the putative function of identified genes at KEGG level 3 suggests that glycolysis/gluconeogenesis, citrate cycle (TCA cycle), pyruvate, starch and sucrose metabolism are key for bacterial survival in the swine microbiome; and were identified in samples from all farms. Similarly, genes involved in metabolic pathways for amino acid metabolism, including alanine, aspartate, glutamate, glycine, serine, threonine, cysteine and methionine were also conserved across all samples and genes with roles in energy and nucleotide metabolism. In addition, the most common pathway among these farms was DNA replication, mismatch repair and homologous recombination associated with replication and repair categories. When we considered genetic information processing, the aminoacyl-tRNA biosynthesis and ribosome pathways were the most common across all farms. ABC transporters and two-component systems were also established as the most common pathway in the environmental information processing categories (Fig. 5).

### Antimicrobial resistome identification harboured in the swine commercial farms based on the caecal microbiome data

**Fig. 6.**
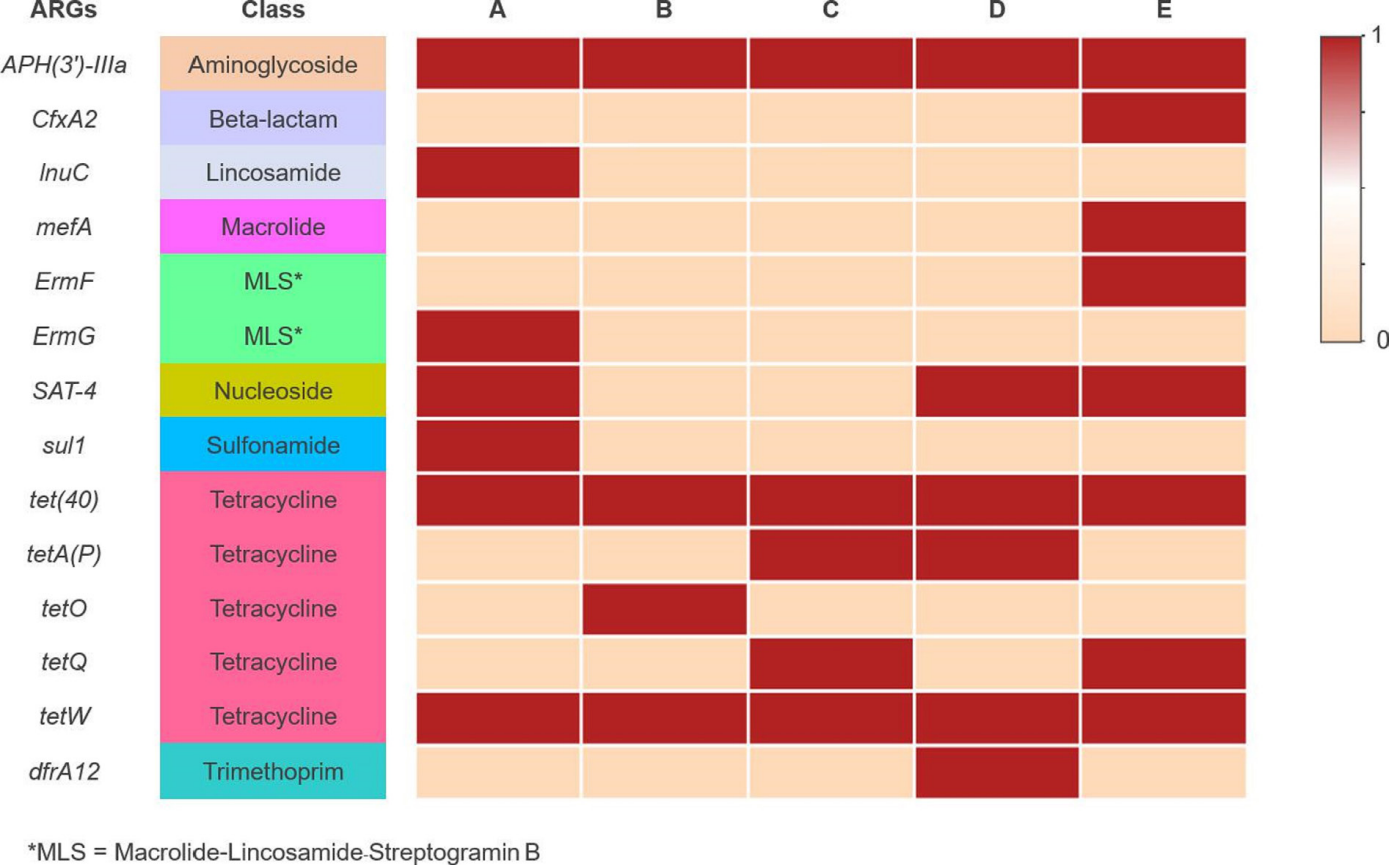
Binary heatmap analysis of antimicrobial resistance genes harboured in the swine caecal microbiome among five commercial farms.

Antimicrobial Resistance Genes (ARGs) were detected through nucleotide comparisons with a curated list of ARGs in the CARD database (The Comprehensive Antibiotic Resistance Database). There were 30 ARGs detected among five caecal microbiomes, which putatively confer resistance against nine antimicrobial classes including aminoglycoside, beta-lactam, lincosamide, macrolide, macrolide-lincosamide-streptogramin B, nucleoside, sulfonamide, tetracycline and trimethoprim (Fig. 6). Genes that confer resistance to tetracycline were the most common ARG, with 15 genes identified from all farms, followed by aminoglycoside resistance (comprised five ARGS) and nucleoside resistance (comprised three ARGs). However, only one gene was found to be resistant to beta-lactam, lincosamide, macrolide, sulfonamide and trimethoprim across all samples. The most common ARGs encountered in all farms were *APH(3’)-IIIa, tet(40)* and *tetW*, which led to aminoglycoside and tetracycline resistance (Fig. 6). For the number of ARGs detected in each farm, there are diverse ARGs in each farm, including farm E, which had the highest abundance of ARGs (eight ARGs), followed by farm A (seven ARGs) and farm D (six ARGs). When focusing on each farm, some ARGs were detected in individual farms, including *InuC, ErmG* and *sul1* in farm A, which conferred to the lincosamide, macrolide-lindosamide-streptogramin B and sulfonamide resistance, respectively. *TetO*, which confers tetracycline resistance, and *dfrA12*, which encoded for trimethoprim resistance, were found in farms B and D, respectively. *CfxA2, mefA* and *ErmF*, associated with beta-lactam, macrolide and macrolide-lincosamide-streptogramin B resistance, were only found in farm E. Furthermore, we realized that some ARGs were shared among each farm, including *SAT-4* linked to the nucleoside resistance, which was shared among farm A, farm D and farm E. *TetQ* was shared by farm C and farm E, whereas *tetA(P*) was shared by farm C and farm D. These two genes were encoded to tetracycline resistance (Figs6  7).

**Fig. 7.**
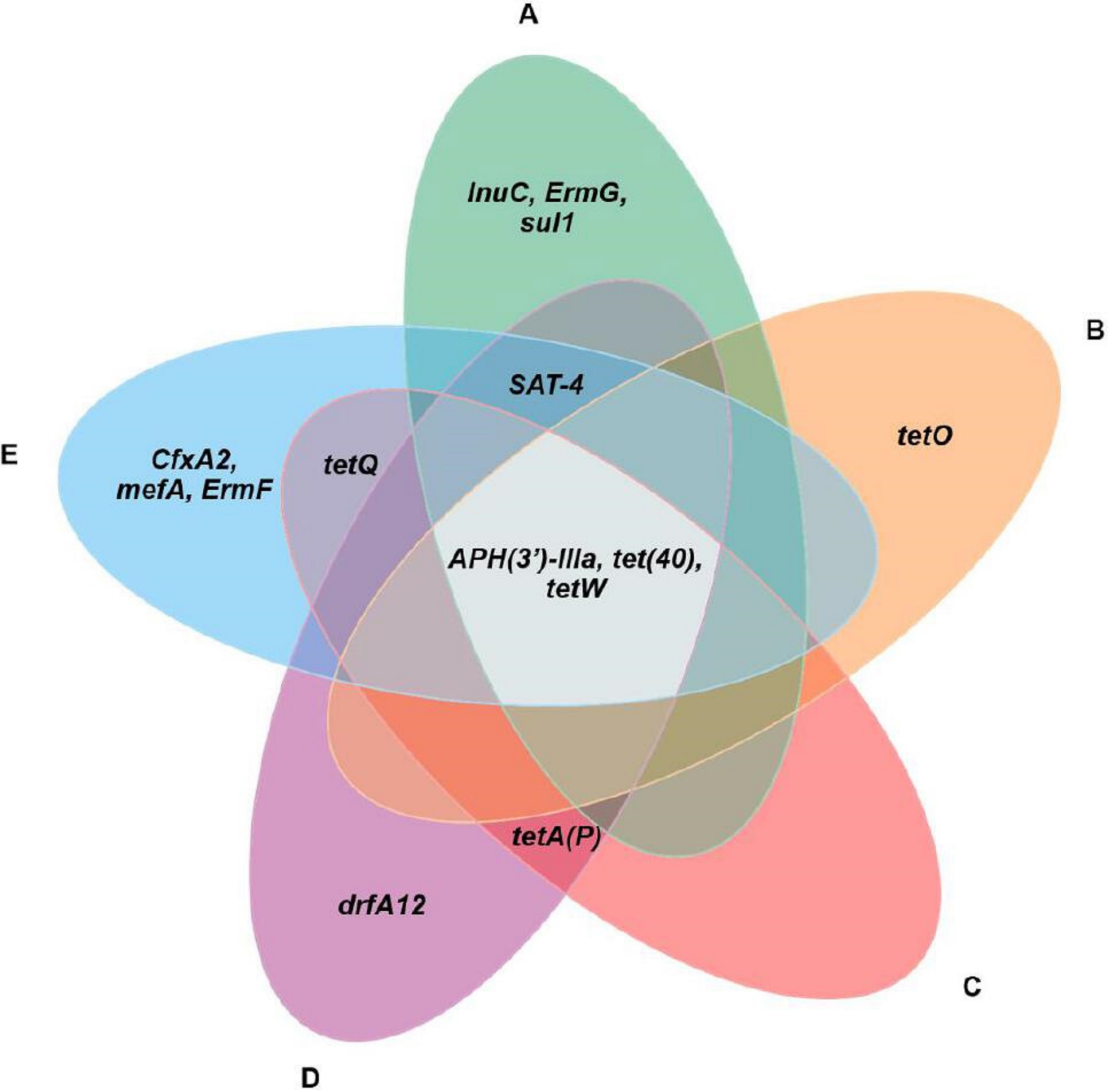
Venn diagram of intersection analysis of antimicrobial resistance genes harboured in the swine caecal microbiome among five commercial farms.

## Discussion

The study incorporated swine caecal samples from five intensive commercial farms located in the upper northern region. The primary objective of this study was to descriptively explore bacterial content and their associated genes from the caecal microbiome of farmed swine. Despite the limited sample sizes, by adopting a shotgun metagenome approach our study expands on the scope of bacterial diversity investigated, incorporating details on genes related to the normal physiology of market-weighted pigs for consumers from multiple bacterial species and genera. Furthermore, we investignste the breadth of antimicrobial resistance genes present in functioning agricultural systems, and their potential to pose a threat to public health.

We used shotgun metagenome sequencing to examine the bacterial diversity in the swine intestinal tracts collected using the intestinal scraping method. While this method is limitated by increased contamination of DNA from the host genome, this method is well-suited for examining micro-organisms that directly interact with the mucosal epithelium. This approach may therefore be useful for addressing questions related to micro-organisms that impact the efficiency of feed utilization in healthy swine. In addition to identification of commensal bacteria typically associated with healthy swine, we identified pathogenic species that can cause illness and physiological changes in the animal’s gastrointestinal tract. These micro-organisms can also be shed into the environment, presenting a potential threat to public health [20,21]. While other, non-bacterial microbial pathogens can also infect farmed pigs [37,38], we did not identify a high proportion of non-bacterial DNA in our samples, however eukaryotic pathogen databases are poorly populated and represents an opportunity that could be reassessed in future studies.

The bacterial composition varied in abundance and taxonomy across different swine farms, potentially due to variation in farm location, size, swine type and feed composition. Antimicrobial usage within the swine farm was associated with the heterogeneity of the bacterial composition found in the swine intestinal tract. Notably, the dominant bacteria differed among farms, including pathogenic, opportunistic and commensal species, which correlate to different properties, both in terms of influencing the host’s physiology and causing disease in both humans and animals. Carriage of multiple species with the ability to cause disease demonstrated that swine could serve as a source of various pathogens beyond foodborne pathogens, which have adverse consequences for the livestock industry and public health [7,8, 39].

The predominant bacterial phyla in the caecal mucosal epithelium samples were *Firmicutes*, *Actinobacteria*, *Proteobacteria* and *Bacteroidota*, which is consistent with previous studies of swine caecal microbiomes [9,19, 40]. *Escherichia coli* was the predominant pathogen found in the caecal microbiome, which is a common pathogen in both humans and animals [41,43]. This pathogen is associated with post-weaning diarrhoea and oedema disease in swine, which can have an enormous economic impact. Moreover, *Escherichia coli* is an important cause of the foodborne illness in humans [44]. The high abundance of *Escherichia* genus is sometimes correlated with antibiotic usage, where it can affect homeostasis in the gastrointestinal tract of animals [8,45, 46]. *Clostridium botulinum* was identified in samples from every farm and commonly found in a variety of environments, such as, in soil, water and the swine gastrointestinal tracts. This pathogen poses a specific food safety risk as its spore’s can survive through processing stages and contaminate food product [47]. The *Corynebacterium* genus was the most common on farm A and is commonly found in animal manure and fertilizer used in agriculture, which can spread to cause human nosocomial infections [48]. Furthermore, high prevalence of antimicrobial resistance are also observed in *Corynebacterium*, potentially correlated with the use of antibiotics in livestock [49,50]. Reports in Europe have helped characterize transmission events between farm workers and swine of specific livestock-associated lineages of *Staphylococcus aureus* [51,54]. The symptoms range from a simple skin infection to the severity of a life-threatening disease among the elderly and immunocompromised people [55]. These findings highlight the potential of swine to harbour disease-causing pathogens and the need for standardized sanitation practices on swine farms.

Commensal bacteria such as *Faecalibacterium*, *Lactobacillus*, *Bifidobacterium* and *Streptomyces*, which are all beneficial for digestion, were identified in all swine caecal samples. *Faecalibacterium prausnitzii* is a commensal bacterium, which is accountable for promoting healthy gut-associated butyrate production, enhancing feed efficiency and microbiome balance [56]. Furthermore, *Bifidobacterium* and *Lactobacillus* the lactic acid bacteria and qualify as probiotics, have the potential to improve feed utilization, and control the balance of pathogenic bacteria in the gut [57]. *Streptomyces* are common soil-dwelling organisms with an extensive ability to produce antimicrobial agents and useful secondary metabolites [58]. Detection of this genus in our microbiome samples suggests a close relationship between the animals and the surrounding environment [59]. *Collinsella* is found in both swine and human gastrointestinal tracts associated with plant-based diets and affects feed intake and efficiency in swine. Although its pathogenicity in swine is unclear, *Collinsella* is linked to important human diseases like irritable bowel syndrome [60,61]. Common bacterial species shared between swine and humans indicates a potential exchange of microbiomes, which may raise public health concerns [12,55].

Metagenome analysis of the swine caecal microbiome also gives us an opportunity to identify specific genes and functional processes that are important in the host–microbe relationship. Using comparisons with the KEGG database, we reconstructed metabolic pathways that were common among the microbiomes of our caecal samples. Functional pathways that were common among healthy swine microbiomes included those associated with processes of nutrient utilization and metabolism, particularly those involved in carbohydrate and amino acid metabolism. Short-chain fatty acids produced through carbohydrate metabolism are essential for good intestinal health and feed efficiency [9,11]. Amino acids like glutamate and aspartate are oxidative fuels of the intestine and are crucial for maintaining intestinal mucosal mass and integrity [62]. ATP-binding cassette transporters and the two-component system facilitate nutrient transport and the use of physiological substrates [9,63, 64]. These findings highlight the significance of the caecal microbiome in swine health and digestion process, however infection with pathogenic species can disrupt microbiome homeostasis and impair digestion [9,65].

Food animals such as swine are regarded as one of the most important antimicrobial resistance pathogen reservoirs, and are exposed to extensive antibiotic usage, via widespread use of antimicrobials in livestock for disease prevention, production efficiency promotion and treatment has led to the emergence of antimicrobial resistance. This global public health concern could result in over 10 million deaths from antimicrobial-resistant bacteria by 2050 if not addressed [66]. Acquiring data from shotgun metagenomics is appropriate for an overview of the antimicrobial resistance genes within one environment, which can provide evidence of long-term antimicrobial usage in the livestock industry [67,68]. The caecal microbiome from all five swine farms contained antimicrobial resistance genes for aminoglycoside and tetracycline resistance, which are commonly used in the swine industry [69]. Various other groups of antimicrobial resistance genes, such as beta-lactam, lincosamide, macrolide, macrolide-lincosamide-streptogramin B, nucleoside, sulfonamide and trimethoprim resistance genes, were also found across the farms. These findings indicate a strong selective pressure resulting from widespread antimicrobial use and highlight the abundance of antimicrobial resistance organisms in industrially farmed swine [70,72]. Rapid industrialization of agricultural processes has diminished physical barriers to recombination, facilitating the spread of AMR genes between species and gene pools [73], often via the exchange of mobile genetic elements and horizontal gene transfer [74,76]. While efforts have been made to prohibit the use of certain antimicrobials for agricultural use, we still identified several ARGs in our swine caecal microbiome samples, consistent with other studies [46,77]. The use of swine manure in agriculture may also be encouraging the dissemination of ARGs from livestock to the environment [78].

Our study provides essential baseline insights into the caecal microbiome within intensive industrial swine farms in the upper northern region of Thailand – an integral area for swine production. However, due to our limited sample size further studies will be required to investigate correlation between modifications to the production environment and swine gut health. The intestinal scraping method used in this study was prone to contamination, with a relatively high number of reads attributed to the host species (*S. scrofa*). This resulted in fewer reads that could be used to interrogate the microbiome; however, the method did allow us to focus on bacteria specifically attached to the caecal epithelial cell [79]. Increased resolution of the microbiome will also facilitate assembly of a broader number of taxonomic groups and individual species. This can provide a window on organisms that have previously been unculturable [80,81]. Functional redundancy in metabolic pathways of organisms’ resident in this very specific niche impairs our ability to segregate and identify beneficial/pathogenic bacterial species [82,83]. Serving as a foundational exploration, this research represents the fundamental information in applying metagenomic analysis to elucidate the diverse range of pathogenic bacteria within the swine digestive tract. Moreover, the investigation of AMR genes was also conducted using the metagenomic approach, presenting the potential for more effective identification of public health concerns. This understanding is pivotal, demonstrating that various types of pathogenic bacteria and antimicrobial resistance genes coexist, potentially spreading through the environment and posing significant public health concerns.

## Conclusion

Our investigation revealed a diverse array of bacteria inhabiting the swine’s caecal epithelium, including pathogens capable of causing diseases in both humans and animals. These findings underscore the potential risk of transmission of these pathogens from swine farms to humans, emphasizing the need for standardized sanitation measures in swine production facilities. We identified commensal bacteria associated with swine feed utilization and efficiency. By leveraging the KEGG metabolic pathway database, we established a link between these bacteria and healthy swine physiology and feed utilization. This understanding can aid in the development of strategies to enhance swine health and optimize feed efficiency. Our study also identified antimicrobial resistance genes within the swine caecal microbiome. This finding raises concerns about the potential impact of historical antibiotic use in swine farms on the prevalence of antimicrobial resistance. Implementing measures to mitigate antimicrobial resistance is crucial for safeguarding public health and ensuring the effectiveness of antibiotics.

## supplementary material

10.1099/jmm.0.001787 Supplementary Material 1.

## Abbreviations

AMR: antimicrobial resistance
ARG: antibiotic resistance gene
CARD: the comprehensive antibiotic resistance database
DNA: deoxyribonucleic acid
KEGG: kyoto encyclopedia of genes and genomes
NCBI: National Center for Biotechnology Information
ORF: open reading frame
TCA cycle: tricarboxylic acid cycle
tRNA: transfer ribonucleic acid
