# Supplementary material for: Commercial farmed swine harbour a variety of pathogenic bacteria and antimicrobial resistance genes
Source: J Med Microbiol. 2024 Jan 17;73(1):001787. doi: 10.1099/jmm.0.001787 (PMC11418424; doi:10.1099/jmm.0.001787)
Supplement: Supplementary Material 1. [file jmm-73-01787-s001.pdf]

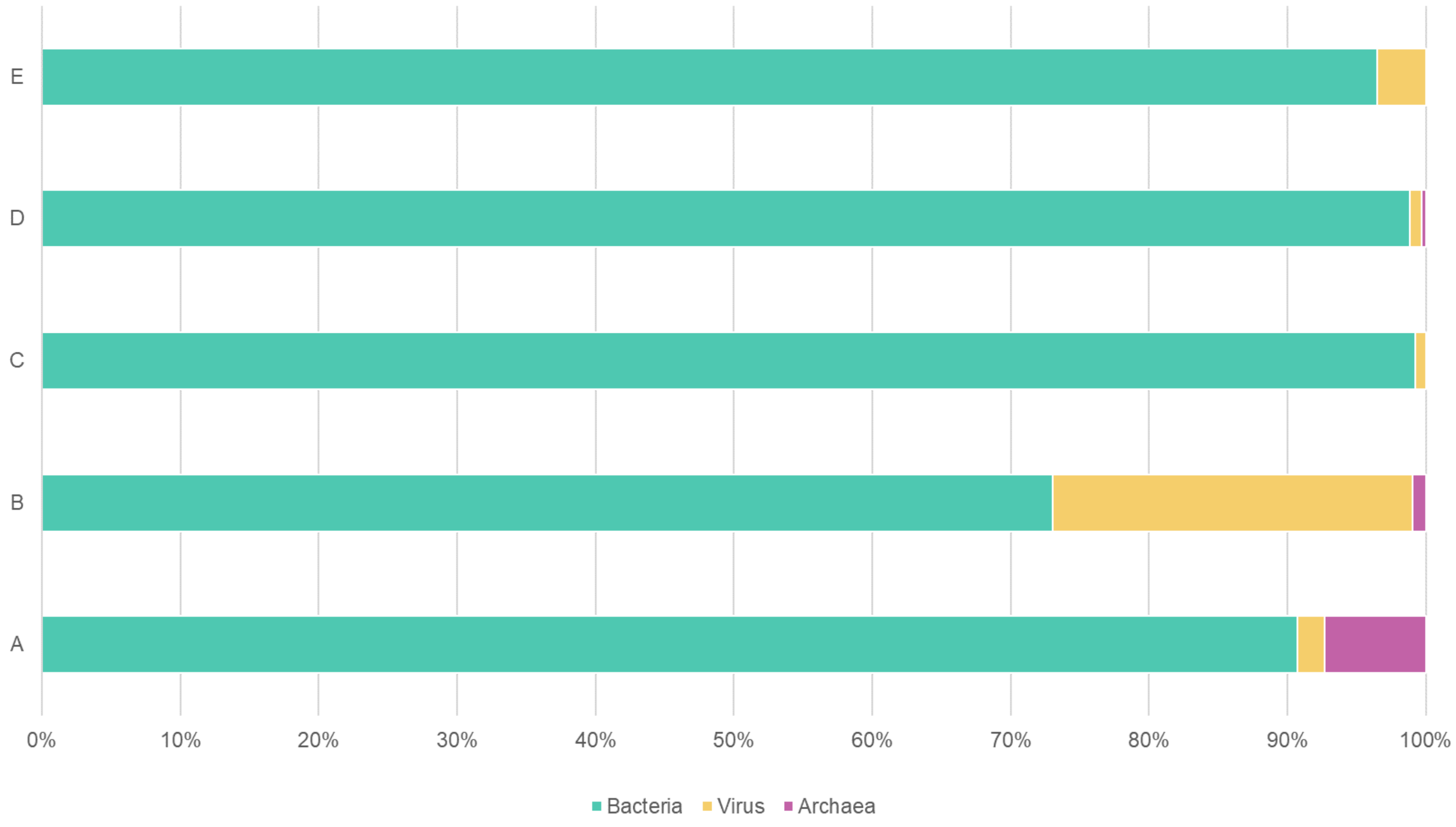

Supplementary Figure S1: The microbial taxonomic composition of the swine caecal microbiome using Kraken2.

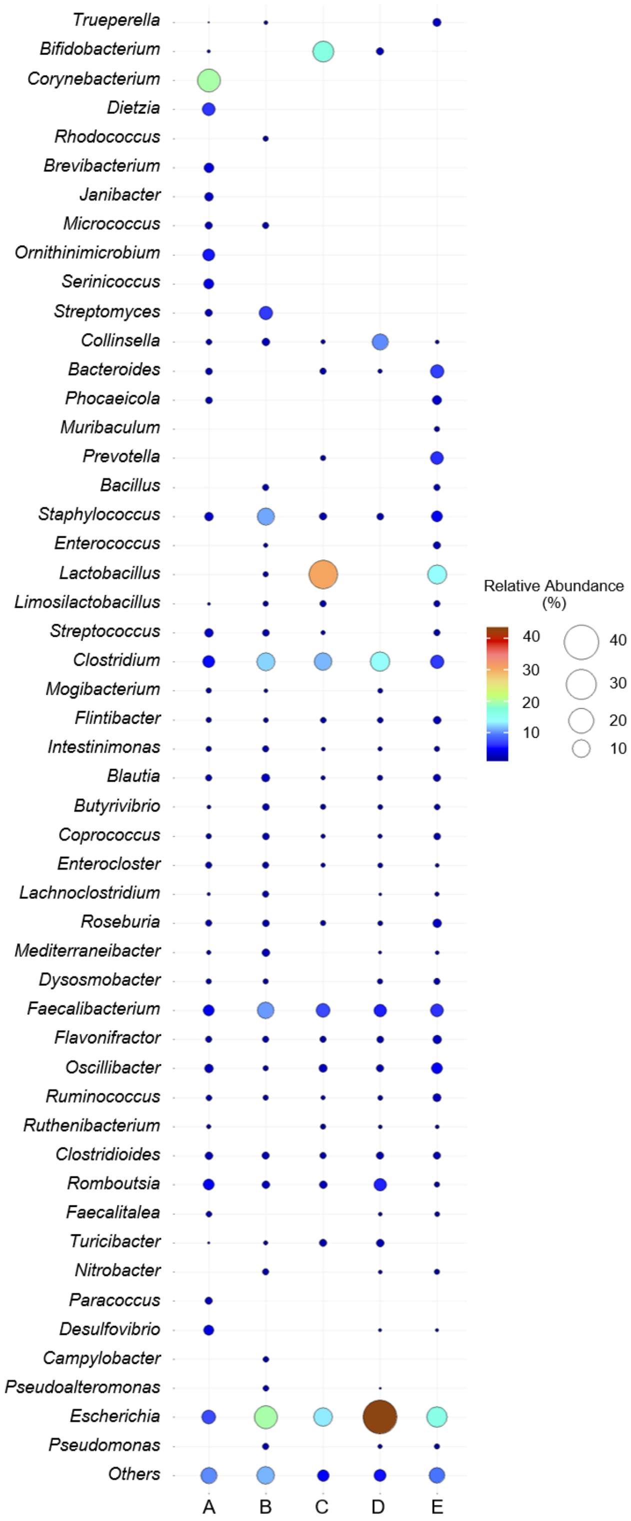

Supplementary Figure S2: The bacterial taxonomic balloon plot of the relative abundance and diversity of the swine caecal microbiome at genus level distributed in each commercial farm. Circle sizes and color represented the percentage of the relative abundance.

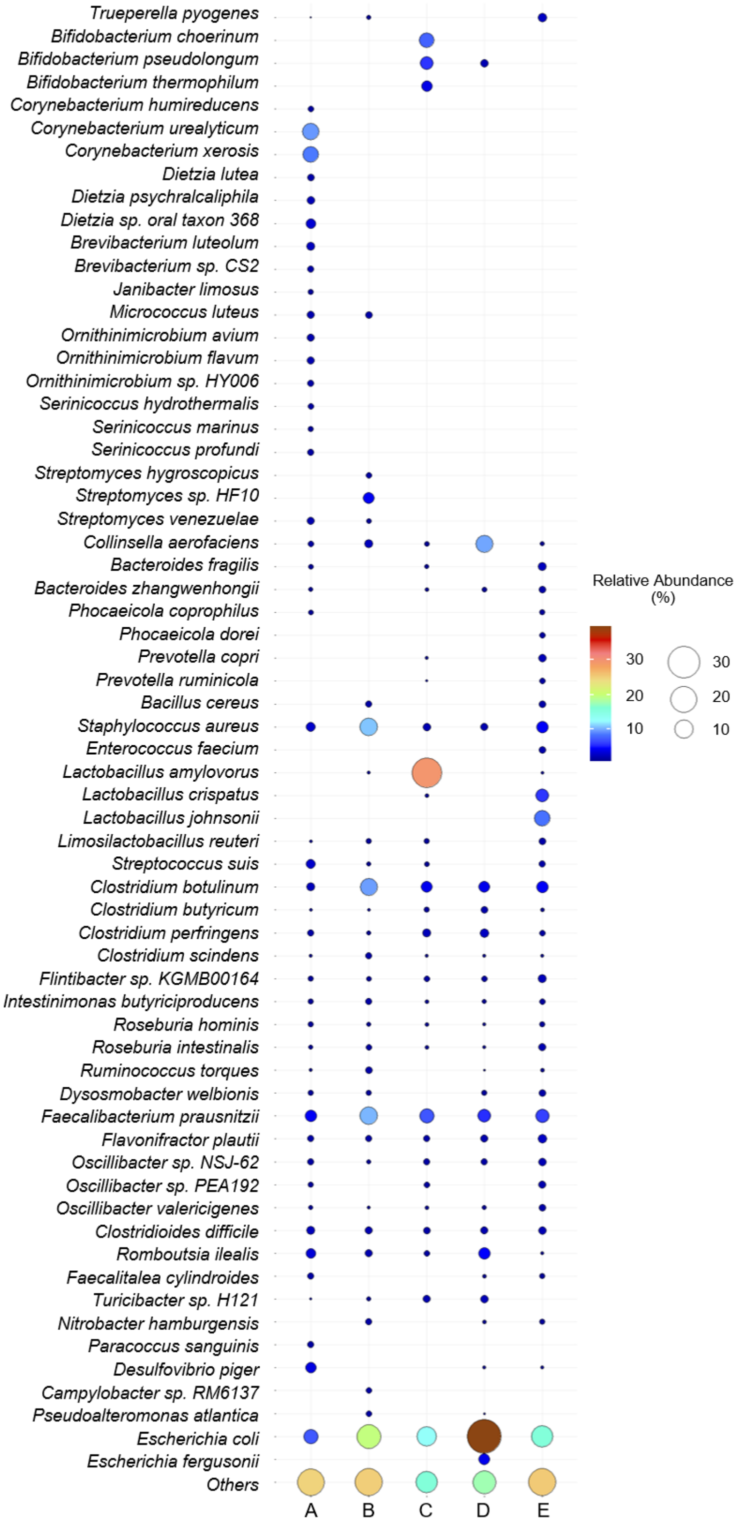

Supplementary Figure S3: The bacterial taxonomic balloon plot of the relative abundance and diversity of the swine caecal microbiome at species level distributed in each commercial farm. Circle sizes and color represented the percentage of the relative abundance.

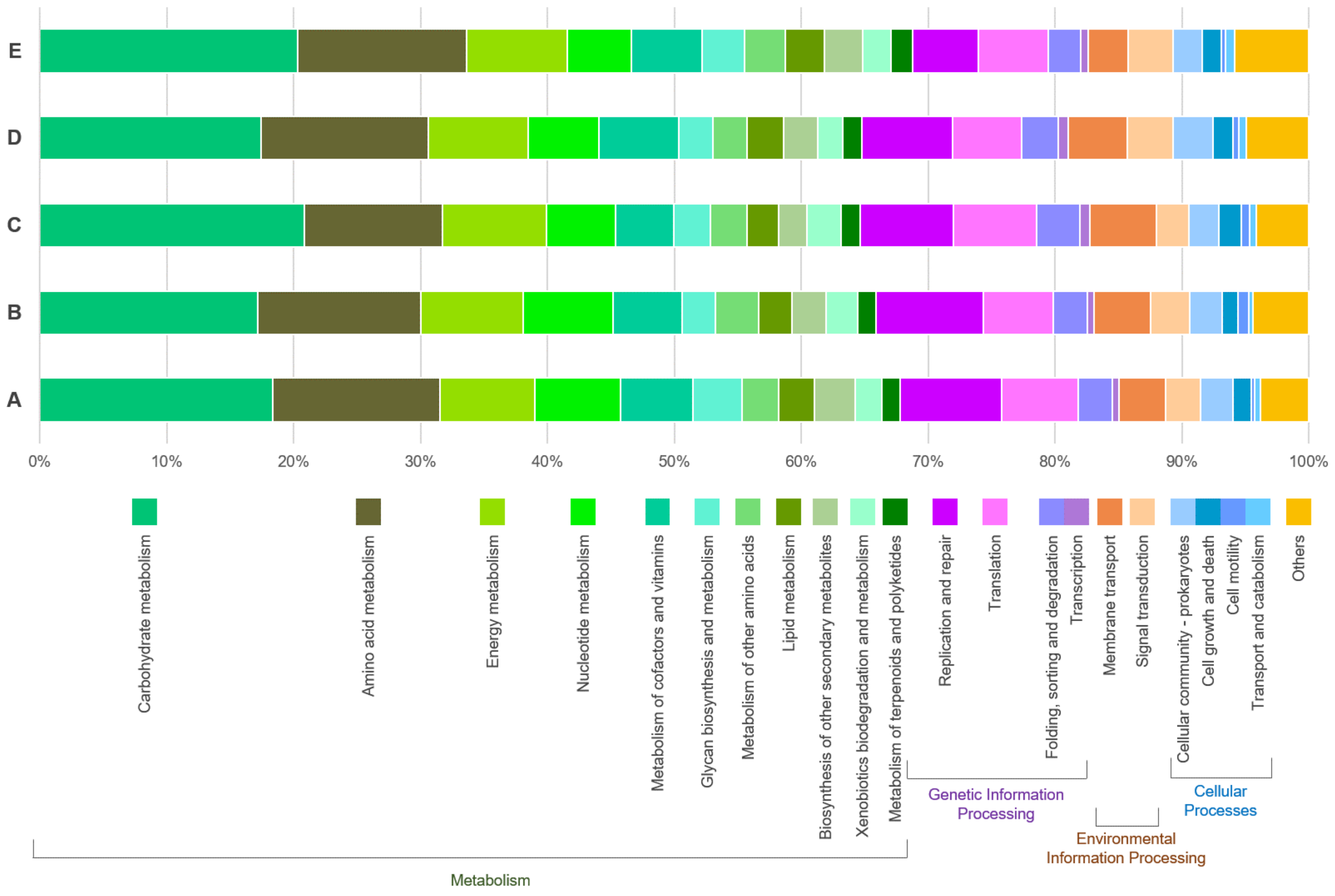

Supplementary Figure S4: Functional characterization of the swine caecal microbiome according to the KEGG annotation pathway database at level 2.

Supplementary Table S1: The summary of the metagenomic sequencing data and statistics for each sample.

Data Production

Sample	Read length (bp)	Raw reads	Quality filtered reads	Host reads	Host removed reads
**A**	150	24,930,615	24,438,189	18,535,664	5,902,525
**B**	150	28,782,351	27,987,909	10,860,533	17,127,376
**C**	150	26,835,788	26,231,972	20,210,619	6,021,353
**D**	150	28,722,734	28,051,350	19,966,498	8,084,852
**E**	150	29,596,965	28,850,725	19,877,608	8,973,117

De novo assembly

Sample	No. of Contigs	Max length (bp)	Min length (bp)	Total length (bp)	N50
**A**	87,529	99,371	500	73,511,030	796
**B**	229,191	43,061	500	167,789,632	705
**C**	65,294	21,314	500	49,138,444	705
**D**	141,142	221,607	500	119,104,342	796
**E**	213,368	34,256	500	188,253,748	834

Gene prediction

Sample	No. of genes	Total gene length (bp)	Average gene length (bp)	% of KO assigned
**A**	58,321	15,099,245	258.90	12.3
**B**	163,943	29,626,285	180.71	3.2
**C**	42,061	8,087,081	192.27	5.8
**D**	90,551	18,220,300	201.22	6.9
**E**	142,151	25,041,046	176.16	3.8

Supplementary Table S2: The individual accession number of the metagenomic sequencing data.

Sample	Accession	Type of Sample	Sequencing Platform	Library Layout
**A**	SRR24680595	Caecal Microbiome	Illumina NovaSeq 6000	Paired end
**B**	SRR24680594	Caecal Microbiome	Illumina NovaSeq 6000	Paired end
**C**	SRR24680593	Caecal Microbiome	Illumina NovaSeq 6000	Paired end
**D**	SRR24680592	Caecal Microbiome	Illumina NovaSeq 6000	Paired end
**E**	SRR24680591	Caecal Microbiome	Illumina NovaSeq 6000	Paired end
